# Prevalence of alternative splicing choices in *Arabidopsis thaliana*

**DOI:** 10.1186/1471-2229-10-102

**Published:** 2010-06-04

**Authors:** Adam C English, Ketan S Patel, Ann E Loraine

**Affiliations:** 1 Department of Bioinformatics and Genomics, University of North Carolina at Charlotte, North Carolina Research Campus, 600 Laureate Way, Kannapolis, NC 28081, USA

## Abstract

**Background:**

Around 14% of protein-coding genes of *Arabidopsis thaliana *genes from the TAIR9 genome release are annotated as producing multiple transcript variants through alternative splicing. However, for most alternatively spliced genes in *Arabidopsis*, the relative expression level of individual splicing variants is unknown.

**Results:**

We investigated prevalence of alternative splicing (AS) events in *Arabidopsis thaliana *using ESTs. We found that for most AS events with ample EST coverage, the majority of overlapping ESTs strongly supported one major splicing choice, with less than 10% of ESTs supporting the minor form. Analysis of ESTs also revealed a small but noteworthy subset of genes for which alternative choices appeared with about equal prevalence, suggesting that for these genes the variant splicing forms co-occur in the same cell types. Of the AS events in which both forms were about equally prevalent, more than 80% affected untranslated regions or involved small changes to the encoded protein sequence.

**Conclusions:**

Currently available evidence from ESTs indicates that alternative splicing in *Arabidopsis *occurs and affects many genes, but for most genes with documented alternative splicing, one AS choice predominates. To aid investigation of the role AS may play in modulating function of *Arabidopsis *genes, we provide an on-line resource (ArabiTag) that supports searching AS events by gene, by EST library keyword search, and by relative prevalence of minor and major forms.

## Background

Most eukaryotic genes contain introns, regions of non-coding sequence that are transcribed into RNA but ultimately removed via a process known as RNA splicing [1]. In alternative splicing (AS), identical transcripts arising from the same locus can undergo multiple splicing programs, in which different segments of the transcribed sequence are removed. The effects on protein sequence and function can be profound [2,3], and there are many examples of genes where AS provides a regulatory mechanism controlling aspects of development and other processes, including flowering in *Arabidopsis thaliana *(reviewed in [4]), sex determination in *Drosophila melanogaster *[5], and aspects of neuronal differentiation in mammals (reviewed in [6]). In *Arabidopsis*, many genes involved in splicing regulation are themselves alternatively spliced and these splicing patterns change in response to diverse treatments. For example, the *Arabidopsis *locus *AT1G16610 *(*SR40*) undergoes cold- and sugar-related shifts in alternative splicing, and the two variants it produces complement different aspects of an *SR40 *mutant phenotype [7]. However, for the vast majority of genes that produce alternatively spliced transcripts, the role that AS plays in modulating gene function is poorly understood.

Several groups have published computational analyses or reviews of alternative splicing (AS) patterns in the *Arabidopsis thaliana *and other sequenced plant genomes (reviewed in [8]). Computational analyses of ESTs and full-length cDNAs suggest that AS is widespread among plant species. However, the overall percentage of genes subject to AS in plants is much smaller than that observed in human, mouse, and chicken genomes, but about the same as in fruit fly and *C. elegans *[9]. Intron retention, a form of AS in which the mature mRNA transcript retains entire introns that are eliminated in other transcripts, accounts for a surprising large proportion (above 30%) of recorded AS events in *Arabidopsis *and rice [10]. Data from whole-genome *Arabidopsis *tiling arrays and quantitative RT-PCR experiments support the theory that for many genes prone to intron retention, the intron-retained forms are expressed in non-trivial amounts [11]. Altogether, these findings may point toward fundamental differences in how splicing mechanisms operate in plants versus animals, where intron retention is rare.

Analysis of gene models predicted from assembled EST and full-length cDNA genomic alignments from rice and *Arabidopsis *discovered that many alternative splicing sites are separated by a small number of bases, typically four or three bases in the case of alternative donor and acceptor sites, respectively [12]. This is also the case in mammalian genomes (reviewed in [13]). Finally, some AS patterns appear to be conserved across different plant species; the gene encoding rubisco activase is one well-known example [14]. Conservation of a splicing pattern suggests that the pattern is under selection and is important for gene function [15].

As noted in [12], alternative donor sites frequently introduce frameshifts, whereas alternative acceptor sites often have seemingly minor effects on the encoded protein sequences, since the alternative sites are usually separated by short distances and occur in multiples of three. This latter observation raises questions about the significance of AS in regulating gene function, since the alternative protein products generated from alternative donor sites are unlikely to differ dramatically unless the splicing difference affects residues that are important for function, such as an active site in an enzyme. Another important question addresses the prevalence of intron retention, which appears to be unusually high in plants relative to animals. One view is that the high percentage of retained intron (RI) events observed in plant EST databases may be due to contamination from genomic DNA or from incompletely processed nuclear RNA.

These studies raise questions about the prevalence and expression of individual splicing variants in plants. What role, if any, does splicing regulation play in defence against pathogens, modulating developmental processes, and adapting to environmental stress? Are all observed variant forms abundantly expressed? For many genes subject to AS, it may be that most transcripts produced at a locus are spliced according to one, dominant pattern, with only a small number of molecules undergoing an alternative splicing pattern. If so, it is possible that most variant forms observed in sequence databases may represent experimental or biological noise, which could be exacerbated by environmental stress or other challenges but even so might not significantly affect phenotype or provide a selective advantage. Alternatively, many variants may appear in a small number of cell types and may represent an aspect of cell-type specific functions. Although splicing patterns may indeed vary, the degree to which this variation is expressed may differ widely between genes, depending on the extent to which individual genes employ AS to modulate gene function in response to stresses in the environment or developmental cues.

In this article, we present a new computational analysis of AS prevalence and expression in *Arabidopsis thaliana *aimed at addressing these questions. We frame our study around the TAIR9 gene models, introduced in the summer of 2009 from the *Arabidopsis *Information Resources (TAIR). The TAIR system uniquely identifies each gene model via an *Arabidopsis *Genome Initiative (AGI) locus code (e.g., AT5G58330) that identifies a region of transcribed DNA together with a suffix of the form .N, where N is a whole number. Each gene model represents a hypothesis about transcriptional regulation of individual genes, including the pattern of splicing, if any, that transcripts arising from the locus undergo. Thus, when multiple gene models are proposed for a single locus, they often represent putative products of alternative splicing.

Following the lead of previously published studies that used ESTs to estimate gene expression levels across diverse samples types [16-18], we assess the frequency with which individual splicing choices proposed as part of the TAIR9 gene models occur in diverse sample types represented by publicly-available *Arabidopsis *ESTs from the dbEST database [19]. To aid in visualization and exploration of the results, we present a database and Web site http://www.transvar.org/arabitag that combines splicing choice frequency analysis with a visualization tool (Integrated Genome Browser [20]) that makes rapid and flexible visualization and inspection of alternative splicing easy to accomplish, even for highly-expressed genes with many thousands of overlapping ESTs.

## Results

### Support for splicing events proposed in *Arabidopsis *gene models

The reference *Arabidopsis *gene models were first created as part of the *Arabidopsis *Genome Initiative and have undergone extensive revision and refinement via manual and automated annotation in which curators assess exon boundaries and splicing events using data from diverse sources, including homology with known protein sequences, co-alignment with ESTs and full-length cDNAs, and, more recently, results from high-throughput proteomics experiments. Thus, each gene model represents a hypothesis regarding splicing patterns, transcription initiation, and transcription termination sites for mature mRNA transcripts arising from a locus.

**Figure 1:**
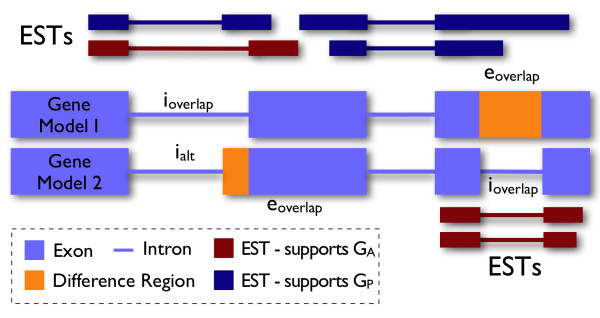
**Recognizing alternative splicing events and EST support**. The diagram presents the genomic alignments for two alternative gene models (shaded purple) alongside alignments for six ESTs. The models propose two AS events, one in which two introns with different boundaries overlap and another in which one model retains an intron that is absent in the other. For each AS event, an exon in one gene model (called e_overlap_) overlaps an intron (labeled i_overlap _) in another gene model; this overlap forms the basis of the AS event detection algorithm described in the Methods section. For the five-prime AS event, which involves an alternative acceptor site, the differentially-spliced intron in Model 1 also overlaps an alternative intron (labeled i_alt _) in Model 2. The orange regions represent Difference Regions (D_R_), segments of genomic sequence that are included in one gene model and not the other. Also shown are ESTs supporting different splicing decisions. For individual AS Events, the gene model that contains the D_R _is called G_P _(Gene Present) and the model that lacks the D_R _is called G_A _(for Gene Absent); ESTs that overlap a D_R _can support either the G_A _or G_P _choice, but not both. In the case of AS events involving retained introns, overlapping ESTs provide support for removal of the intron when their boundaries coincide with the intron's boundaries. Alternatively, they provide support for retention of the intron when they contain a block of alignment that begins 20 bases on either side of the intron boundary and extends at least 20 bases within it.

As of July 2009, the dbEST database contained around 1.5 million *Arabidopsis *ESTs sequenced from 183 libraries. We aligned these to the TAIR9 genome and filtered the alignments for quality and for evidence of splicing, yielding nearly 500,000 ESTs that showed evidence of having been spliced (Table 1). Compared with publicly available EST libraries from other species (especially human and mouse) the total number of available ESTs for *Arabidopsis *is small, and so it is likely that many introns and splicing events proposed as part of the TAIR9 gene models may not be well-represented among the currently available ESTs. To assess this, we first examined the degree to which the spliced ESTs available from dbEST provide experimental support for introns proposed within the TAIR9 gene models. For each intron in the reference gene model collection, we counted the number of ESTs in the spliced EST data set that contained a gap that aligns with the intron's proposed boundaries. (An example diagramming EST support for a splicing pattern appears in Figure 1.) We found that most introns were indeed supported by at least one EST, but around 40% (49,073 of 121,699 introns proposed in 27,118 multi-exon TAIR9 gene models) lacked support among the spliced ESTs data set.

**Table 1:** Arabidopsis ESTs aligned onto the TAIR9 genome sequence.

Data Set	No. ESTs
*A. thaliana *ESTs from dbEST	1,527,298

ESTs that map to one location in the genome	1,243,950

ESTs that exhibit splicing	479,896

ESTs were downloaded from dbEST and aligned onto the *Arabidopsis *genome using blat. ESTs that aligned with at least 95% identity and 90% coverage and aligned exactly once under these quality criteria were kept. Next, ESTs with alignments that showed evidence of splicing were used to create the intronEST data set, which was then used to examine EST support for splicing choices proposed as part of the *Arabidopsis *TAIR9 gene models.

**Figure 2:**
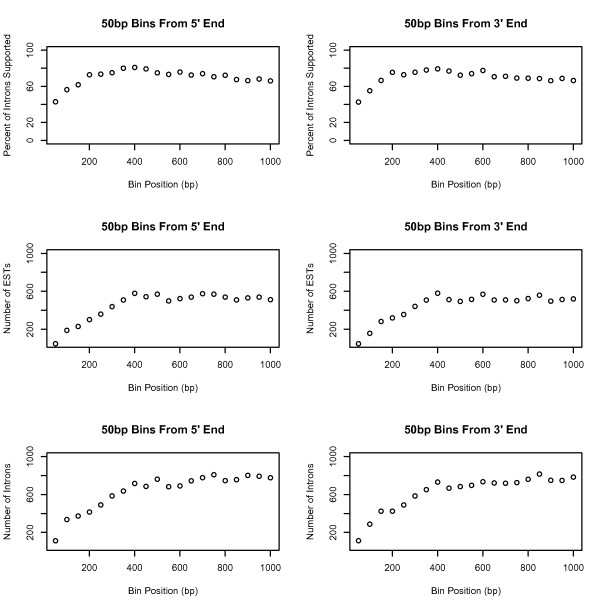
**Intron support and intron position**. The diagram shows the percentage of supported introns among introns removed from sequential 50 base pair regions with respect to the spliced transcript, for spliced transcripts 2,000 bases or larger.

We next investigated possible biases between intron support and other characteristics of introns, including size, splice site consensus sequence, and position within the spliced transcript. Most but not all annotated introns in *Arabidopsis *contain five and three prime splice boundary consensus sequences GT and AG. Since many EST alignment tools (such as blat [21]) take advantage of splice site consensus sequences to maximize speed and accuracy, some introns that deviate from the expected patterns might be missed. However, we found no enrichment of unsupported introns among the population of introns with non-canonical boundaries. Nor did we find any bias with respect to size of introns and their level of support. Another source of bias may arise from the position an intron occupies within the unspliced, primary transcript. EST projects often aim to obtain the five prime end sequences of transcripts in order to characterize protein-coding regions. However, the *Arabidopsis *ESTs derive from many different sequencing and library construction methods, including pyrosequencing via 454 GS/FLX [22,23], sequencing of RT-PCR products [24], and traditional Sanger sequencing of cDNA clones [25,26]. Thus, it is possible that when considered as an entire population, biases with respect to intron position within a transcript and level of EST support may not be significant. To assess this, we examined the level of EST support for introns spliced from discrete regions distant from the five and three prime ends of the spliced transcript (Figure 2). We observed that introns nearest the ends of transcripts (removed from within the first 100 bases) tend to be less-well-supported than introns located within the next 100 bases, but overall, there was not much bias with respect to distance from the five or three prime ends of transcripts.

### AS events proposed in gene models

**Figure 3:**
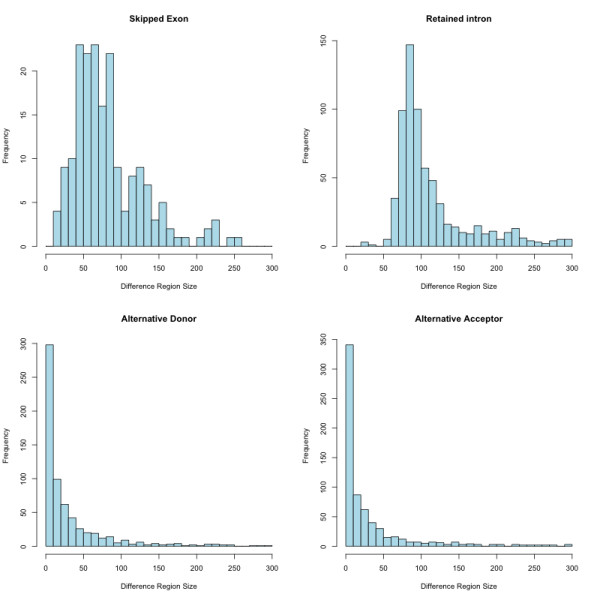
**Size distribution of Difference Regions in the TAIR9 gene models**. The size distribution for Difference Regions in which each AS choice is supported by one or more ESTs is shown for AS events involving exon skipping (top left), retained introns (top right), alternative donors (bottom left) and alternative acceptor sites (bottom right.) Not shown are five retained intron Difference Regions larger than 600 bases.

We next examined alternative splicing events proposed as part of the TAIR9 gene models. For each pairwise combination of gene models arising from the same gene, we searched for Difference Regions, places where an exon in one model overlapped an intron in another, adapting a previously published method [3]. The region of overlap (designated D_R_, for Difference Region) represents a segment of sequence that is included in one model (termed G_P _for gene present) but is absent from another (termed G_A _for gene absent). Excluding all differences not arising from alternative splicing (e.g., alternative promoters or polyadenylation site choice) and removing redundancies arising from genes with more than two gene models yielded 5,891 unique Difference Regions affected by alternative splicing in the TAIR9 gene models. Figure 3 presents histograms showing the Difference Region size distributions for different categories of AS events, where both forms were supported by at least one spliced EST. The median size for Difference Regions not involving retained introns (non-RI) is 17 bases, and 25% of the non-RI Difference Regions is smaller than 5 bases. The median size for retained intron (RI) Difference Regions is 97 bases and more than 95% of the RI Difference Regions is larger than 60 bases. One consequence of the generally smaller size for the non-RI Difference Regions is that biases favoring the G_A _or G_P _forms due to location of splice sites relative to the ends of the affected transcripts will likely be small, since the alternative sites are rarely separated by large distances.

### EST support for proposed AS events

We next assessed the degree of support for each alternative splicing choice for each Difference Region. For this, we tabulated the spliced EST alignments that supported either i_alt _or i_overlap _(for non-RI events) or that supported e_overlap _or i_overlap _(for RI Events.) To detect possible bias toward one or the other mutually exclusive alternatives, we considered each informative EST as a single Bernoulli trial. For each unique Difference Region for which there was at least one supporting EST, we tested the null hypothesis that there is no bias for or against either variant form, i.e., either splicing alternative was equally likely. Altogether, there were a total of 4,909 independent tests, of which 35% produced values less than or equal to 0.05, the generally accepted alpha level for statistical significance. If the data were entirely random, we would expect that around 5% of the tests would yield p values below the 0.05 alpha level purely due to chance, a consequence of multiple hypothesis testing. Therefore, we adjusted the alpha level downward using the highly conservative Bonferroni correction, requiring that individual tests yield p values less 1.02 × 10^-5 ^(alpha divided by the number of tests) to achieve statistical significance. Even with this highly conservative adjustment, we observed significant bias in splice site choice in more than 14% of the Difference Regions.

This statistical testing framework allows for identification of biased splicing only when there are enough informative ESTs to rule out the possibility that each choice is equally likely. The remaining Difference Regions with p values larger than the critical value may represent alternative splicing choices in which there is indeed a strong preference for one choice, but a paucity of available ESTs does not support ruling out the null hypothesis. For example, a Difference Region with just one informative EST would yield a p value of 1, because there is not enough data to assess any possible bias. Also, the deviation from equal probability between choices may not be very great; for many AS events, there may be a dominant isoform, but the other form may also be well represented.

**Figure 4:**
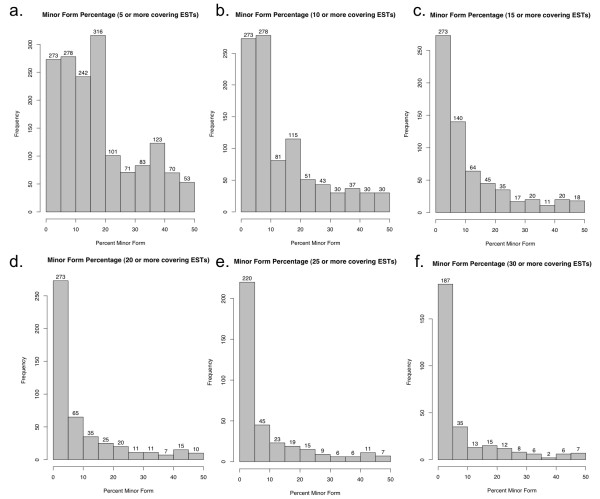
**Histograms showing the distribution of percent minor form among alternative splicing events in Arabidopsis**. Percent minor form is calculated as the number of ESTs supporting the less frequently observed choice divided by the total number of ESTs supporting either choice, multiplied by one hundred. Only AS events were counted for which there was at least one EST supporting each alternative and where at least (a) 5 (b) 10 (c) 15 (d) 20 (e) 25, or (f) 30 ESTs overall support both forms together.

We therefore assessed the magnitude of AS choice preference observed among AS events. We first examined Difference Regions for which there were 30 or more ESTs (event support cutoff >=30), reasoning that 30 overlapping ESTs should provide a large enough sample size to assess whether the ESTs exhibit significant skew toward one splicing alternative, when all the data are considered together. To rule out cases where the TAIR9 gene models might erroneously propose a splice boundary that simply may not occur in nature, we also required that each choice be supported by at least one overlapping EST. We found that among 291 AS events with 30 or more covering ESTs and where at least one EST supported each choice, there was a surprisingly large majority (76%, 222) in which the minor form was represented in 10% or less of the overlapping ESTs (Figure 4F). Figure 4 also shows the distribution of percent minor form with smaller support cutoff values. Even when we required only five or more ESTs to overlap a Difference region, we observed a distinct skew between minor and major forms.

Although the majority of AS events clearly favor one dominant choice, there are still a small but significant number of AS events where both choices are reasonably well-represented among the ESTs, suggesting that these alternative forms may be co-expressed in some of the same cell or sample types. A list of these appears in Table 2. Table 2 lists 22 alternatively spliced genes and 23 Difference Regions for which the ESTs were close to evenly divided in that the minor variant frequency was between 30 and 50%. We examined these using the Integrated Genome Browser in combination with the ArabiTag splice-mining system developed as a companion database and query interface for this paper. One of these encodes rubisco activase, previously mentioned as a well-documented example of AS in plants [14]. The rest encode genes with diverse functions, including RNA processing; translation; intracellular transport; fatty acid biosynthesis; photorespiration, and targeting to the chloroplast. In all cases, these genes likely represent examples of genes where AS is reproducible and robust across diverse sample types and unlikely to be an artifact arising from experimental error or data processing. Among the Difference Regions described in Table 2, nearly half (eleven) involve changes in the untranslated regions of the spliced products. And of the remaining Difference Regions located in translated regions, most are small, typically involving three bases.

**Table 2:** Genes with AS events covered by at least 30 ESTs and where each choice is about equally supported.

Locus	Abbreviated Description (TAIR)	**G**_**A**_	**ESTs G**_**A**_	**G**_**P**_	**ESTs G**_**P**_	AS type	**Size D**_**R**_	% MF
AT5G36290	Uncharacterised protein family UPF0016 (InterPro: IPR001727)	.1	30	.2	16	RI	100 (U)	34

AT5G62000	Encodes an auxin response factor. Mutants have ... defects including enlarged rosette leaves, reduced fertility, later senescence, hypocotyl elongation defects, enlarged seeds and enlarged cotyledons... Increase in seed size due to increased cell proliferation.	.2	16	.3	20	AS	3 (U)	44

AT5G58330	malate dehydrogenase (NADP), chloroplast, putative; FUNCTIONS IN: oxidoreductase activity	.2	33	.1	62	AS	3 (P)	35

AT5G66240	transducin family protein/WD-40 repeat family protein; FUNCTIONS IN: nucleotide binding; LOCATED IN: CUL4 RING ubiquitin ligase complex	.1	13	.2	25	RI	89 (P)	34

AT5G64200	encodes an SC35-like splicing factor ... localized to the nuclear specks.	.1	10	.2	22	RI	190 (U)	31

AT4G35450	Involved in targeting of chloroplast outer membrane proteins to the chloroplast. Double mutants of AKR2A and the highly homologous AKR2B have yellow leaves, significantly reduced chloroplast proteins, and no thylakoid membranes.	.1	32	.3	39	DS	44 (U)	45

AT4G36690	ATU2AF65A; FUNCTIONS IN: RNA binding, nucleotide binding, nucleic acid binding; INVOLVED IN: nuclear mRNA splicing...defence response to bacterium	.1	21	.2	17	RI	273 (P)	45

AT4G30760	Unknown	.2	19	.1	26	AS	3 (P)	42

AT4G16162	CONTAINS InterPro DOMAIN/s: Leucine-rich repeat (InterPro:IPR001611); BEST A. thaliana ... match is: serine/threonine protein kinase-related	.2	15	.3	15	ES	58 (P)	50

AT3G23830	encodes a glycine-rich RNA binding protein. Gene expression is induced by cold and reduced by ionic (salt) and non-ionic (mannitol) osmotic stress. Lines overexpressing the gene are slightly more tolerant to osmotic stress during germination.	.2	21	.1	17	AS	12 (U)	45

AT3G01540	RNA HELICASE DRH1	.1	28	.2	32	AS	3 (P)	47

AT3G62840	LOCATED IN: small nucleolar ribonucleoprotein complex, nucleus; CONTAINS InterPro DOMAIN/s: Like-Sm ribonucleoproteinBEST *A. thaliana *protein match is: small nuclear ribonucleoprotein D2, putative/snRNP core protein D2,	.2	16	.1	18	AS	3 (P)	47

AT2G05990	Encodes enoyl-ACP reductase a component of the fatty acid synthase complex. A reduced function mutation in this gene, mod1, was found in a screen for premature cell death mutants. Mutant plants have reduced lipid level and pleiotropic morphological defects, including chlorotic and abnormally shaped leaves	.2	24	.1	33	AS	3 (U)	42

AT2G19730	60 S ribosomal protein L28 (RPL28A)	.2	63	.1	67	AS	4 (U)	49

AT2G17442	Unknown	.4	13	.1	24	AS	3 (P)	35

AT2G17442	Unknown	.4	23	.3	12	DS	31 (U)	34

AT2G39730	Rubisco activase, a nuclear-encoded chloroplast protein that consists of two isoforms arising from alternative splicing in most plants.	.1	201	.2	187	AS	11 (P)	48

AT1G64750	Unknown	.1	18	.2	20	RI	90 (U)	47

AT1G67700	Unknown	.2	22	.1	18	DS	14 (P)	45

AT1G69510	Unknown	.1	22	.3	13	AS	5 (U)	37

AT1G71470	Unknown	.2	21	.1	9	AS	9 (P)	30

AT1G80380	encodes a glycerate kinase which catalyzes the last step of photorespiration C2 cycle	.2	26	.3	13	DS	57 (P)	33

AT1G26850	dehydration-responsive family protein; LOCATED IN: Golgi apparatus, membrane; CONTAINS InterPro DOMAIN/s: methyltransferase putative	.2	9	.3	21	DS	64 (U)	30

Functional descriptions are from the version of the TAIR Web site. Columns labelled G_A _and G_P _indicate which gene models (using TAIR suffixes) correspond to the Gene Absent and Gene Present variations, respectively. ESTs G_A _and ESTs G_P _contain the number of spliced ESTs supporting the G_A _or G_P _splicing pattern. AS type refers the type of alternative splicing involved in the AS event. RI means retained intron; AS means alternative acceptor site (3-prime boundary of an intron); DS means alternative donor site (5-prime boundary of an intron); ES means exon skipping. The column titled Size D_R _gives the size of the Difference Region and whether it changes protein-coding sequence (P) or untranslated (U) region of the gene models. The last column (titled % MF) gives the percentage of overlapping ESTs (ESTs G_A _and ESTs G_P_) that support the less commonly observed (minor) form.

When we examined Difference Regions size trends overall, we found that the highly skewed AS events tended to have larger Difference Regions than the less skewed AS events. For example, the median Difference Region size for the AS events in which the minor form accounted for fewer than 10% of the ESTs was 61 bases, while the median size for the Difference Region for AS events where the minor form was better-represented was only 18 bases. A Wilcoxon rank sum test of assessing the Difference Region size differences between the less skewed versus highly-skewed AS events regions yielded a p value of 4 × 10^-7^, providing additional support illustrating the observation. Thus, in general, AS events with minimal bias toward one variant at the expense of the other tend to involve shorter Difference Regions than do AS events with a strong preference toward one form.

### Test for cell type-specific splicing patterns

The 70% of AS events for which ESTs showed skew toward one splicing choice may include some AS choices that are never or rarely utilized in the cell types where the gene is expressed. However, it may instead be the case that for at least some of these, the less-frequently-observed variant may actually be the dominant form in some small subset of cells that was underrepresented in the bulk of the samples. It is extremely difficult to distinguish these possibilities without access to ESTs prepared from a single cell type. However, we were able to begin addressing this issue using one large library in the collection that was prepared from a single cell type: ovules dissected from flowers [23].

The spliced ESTs from the ovule library provided support for AS events involving 681 distinct Difference Regions. Of these, the library contained support for both G_A _and G_P _choices for only twelve Difference Regions. These twelve Difference Regions represent candidate genes where alternative splice variants may co-occur in the same cell type or even in the same cells - ovules, in this case. We then asked if the ovule library data contained any examples of ovule-specific splicing where the dominant isoform appearing in the ovule library was different from the dominant isoform appearing in other libraries. In other words, we looked for Difference Regions covered by ESTs in the ovule library that seemed to "buck the trend" by favoring a different major form when compared to ESTs from other libraries. Examples of this type might therefore represent splicing patterns that occur primarily in ovules and could be candidates for cell-type specific splicing in *Arabidopsis*. For this analysis, we examined Difference Regions in which both the ovule library and the comparison libraries contained at least three overlapping ESTs supporting either G_A_, G_P_, or both. For the comparison, we used several dbEST libraries with the same or better sequence coverage within the spliced EST data set, calculated as the summed size of all ESTs in the spliced EST data set. We found no AS events where the dominant isoform in the ovule library was not also the dominant isoform overall. However, there were only 139 AS Events that were supported by at least three ovule ESTs. By contrast, the most diverse collection (with respect to splicing) comes from dbEST library id 20522, derived from "*Arabidopsis *roots, inflorescence, callus, young seedlings and *Arabidopsis *treated with cold, heat, salt, 2,4-D, hydrogen peroxide, UV, IAA, *Xanthomonas *and *Pseudomonas *[24]." There were nearly 700 AS Events that were supported by three or more ESTs this highly heterogenous collection. However, it is possible that if further sequencing were performed from the ovule library, ovule-specific splicing events could be found.

### Support for retained intron events

A large percentage (around 30%) of AS events proposed in the reference *Arabidopsis *gene models involve retained introns (RI), in which some gene models retain introns that are removed in other models associated with the same gene. We therefore asked to what degree we could observe support for retained intron events in the ESTs collection. However, the possibility of contamination from genomic DNA sequences among the ESTs collection presented difficulties in that an EST that co-aligns with a retained intron might have arisen from genomic DNA contamination affecting the source cDNA libraries. Therefore, we addressed intron retention prevalence by examining support for retained introns among the intron-EST data set, which show evidence of splicing and therefore are more likely to represent expressed, fully-processed mRNA sequences.

The annotated gene models included 2,119 proposed retained introns alternative splicing events. Of these, there were 713 RI events in which both forms (retained intron and spliced intron) were supported by at least one spliced EST. For each of these proposed RI Difference Regions with spliced EST support for both forms, we examined the level of EST support for the retained intron (G_P_) pattern or where the intron was removed (G_A_). Out of the 713 RI Difference Regions where at least one EST supports both variations, we found 414 Difference Regions in which the G_A _(removed intron) form was the dominant form and 209 Difference Regions in which the G_P _(retained intron) was the dominant form. In some cases, the support for intron retention was quite dramatic; for example, the intron-retained form of AT2G21660, which encodes a glycine-rich RNA binding protein, was supported by over two hundred overlapping ESTs, but the intron-spliced form (G_A_) was support by only one overlapping EST. In this case, the intron-retained form predominates.

### ArabiTag: an on-line tool for assessing splicing diversity by library

Understanding the diversity of splicing choices and their expression within individual cell or sample types is critical to studying how these choices affect gene function. To make it easier to assess whether a given gene undergoes cell type-specific or condition-specific splicing patterns, we developed ArabiTag, an on-line tool that assesses and reports EST evidence for individual alternative splicing choices proposed as part of the TAIR9 gene models. Although there are many on-line tools that make it possible to use ESTs to identify AS patterns, including both the TAIR and the dbEST Web sites, ArabiTag is the only tool aimed at assessing the relative abundance of splicing choices represented in available ESTs.

**Figure 5:**
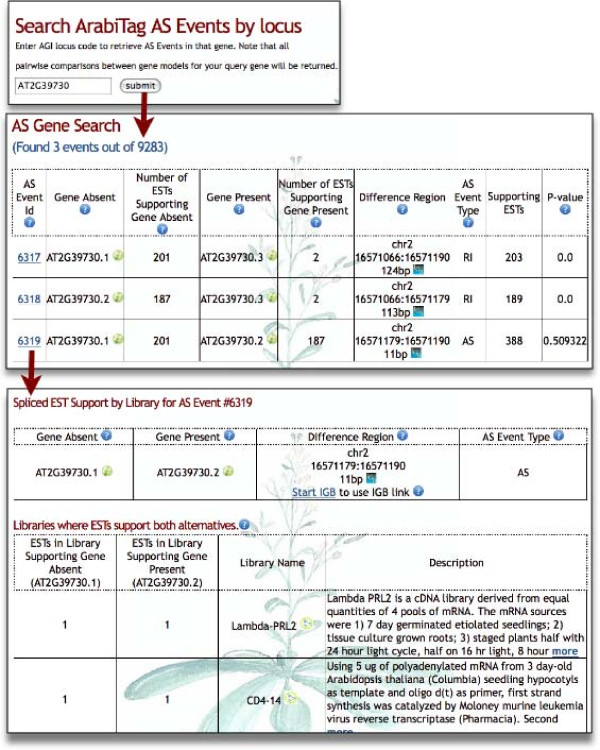
**ArabiTag on-line tool for assessing frequency of splicing choices from Arabidopsis gene models**. Screen captures showing query and results pages from ArabiTag http://www.transvar.org/arabitag are shown. Searching for locus id AT2G39730, encoding rubisco activase, a well-known example of conserved AS in plants [14,34], retrieves three AS Event ids and their corresponding Difference Regions. The alternative splicing choices for the third AS event on the list are supported by multiple ESTs. Clicking the AS Event id link opens an AS Report page in ArabiTag that shows the breakdown of support for each choice, by library, in three different tables according to which choice the ESTs support. Libraries that contain a mix of different ESTs that support both choices appear in the first table, libraries with ESTs that support just one alternative (G_A_) appear in the next table, and libraries with ESTs that support the other alternative (G_P_) appear in the final table. In all cases, the number of ESTs from each library that support G_A _or G_P _are also listed, thus providing the user with information about possible tissue or sample-type specific alternative splicing. However, in this case, both rubisco activase forms are widely expressed, suggesting they may co-occur in the same cells or cell types.

The ArabiTag tool features query pages that allow users to look up alternative splicing events by gene model AGI code and then view a summary of ESTs from different libraries that support each splicing choice. An example appears in Figure 5, showing ESTs that support splice variants for the gene encoding rubisco activase (AGI code AT2G39730), one of the earliest characterized examples of AS in *Arabidopsis *[14]. For completeness, ArabiTag classifies each alternative splicing choice made between every pair of individual gene models as single AS Events. Thus, when there are several models associated with a locus, some AS Events refer to the same Difference Region. Although the results as reported may seem unnecessarily redundant at first glance, we have found that reporting the results for each combination of gene models makes it easier to assess and evaluate all the AS choice differences that occur at a locus.

Once the tool has returned a listing of AS Events, users can then view a report listing each library that contains one or more ESTs supporting the AS Event. The AS Event report page (Figure 5) breaks down EST support into three tables: the first table, appearing at the top of the page, lists libraries that contain a mix of ESTs that support both splicing choices associated with the AS Event. The second table lists libraries where ESTs support just the G_A _splicing variation, and the third table lists libraries in which ESTs support just the G_P _splicing variation. To our knowledge, no other splicing analysis tool provides this level of detail regarding the prevalence of AS choices among different EST collections and, in theory, could make it possible for users to discover condition or stage-specific alternative splicing in *Arabidopsis*. To aid such analysis, the tool also supports downloading of query results in a simple comma-separated format designed for imports into data analysis programs such as R or Excel.

**Figure 6:**
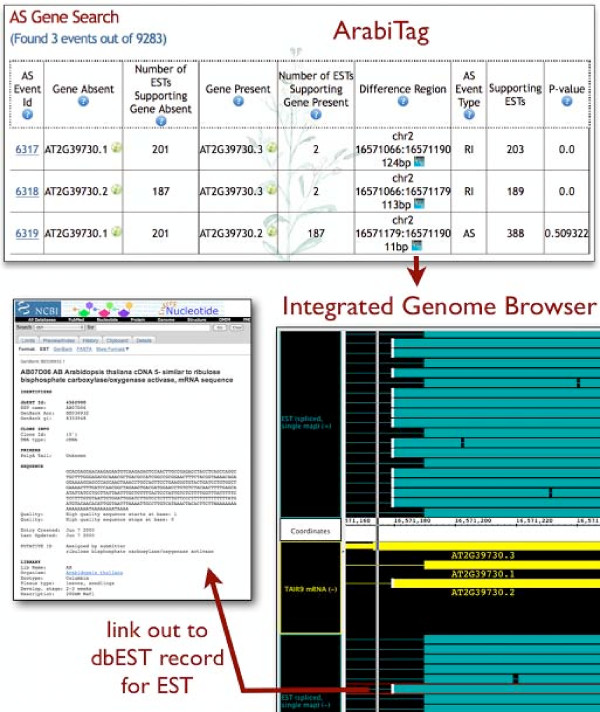
**ArabiTag linking to Integrated Genome Browser**. Screen captures showing query and results pages from ArabiTag http://www.transvar.org/arabitag are shown. A page describing alternative splicing of AT2G39730 (encoding rubisco activase) offers an "IGB link" icon that, when clicked, directs the currently running instance of the Integrated Genome Browser to display the region specified in the IGB link. For convenience, the page also includes a link to the on-line launch page for the IGB software http://www.bioviz.org/igb. To use the IGB link, users download and launch a copy of the IGB software, load the spliced EST and TAIR9 mRNA data sets from the BioViz DAS2 and BioViz Quickload data sources, setting the load mode for the TAIR9 mRNA data set to "Whole Genome." Clicking the IGB link within the ArabiTag Web page directs IGB to zoom and scroll to the Difference Region for this AS choice. Clicking the "Refresh Data" button instructs IGB to retrieve all the spliced ESTs that overlap the region, allowing the user to compare their boundaries with the TAIR9 gene models. Clicking an EST (as shown in the image) activates an edge-matching function, where all items with boundaries identical to the selected item (an EST in this case) acquire an edge-matching icon - a white bar drawn on top of the matching boundary. Right- (or control-clicking) the EST offers the user the option to visit the NCBI Web site and view the dbEST record for the selected EST, revealing it comes from a library prepared from salt-stressed seedlings.

Once a user has decided to focus attention on a single AS event and its associated Difference Region, he or she can then use the Integrated Genome Browser to view the region and inspect the overlapping gene models and ESTs (Figure 6). The IGB program is available via a Java Web Start download page at http://www.bioviz.org/igb. To view Arabidopsis data and the TAIR9 protein-coding gene models, users select species *Arabidopsis thaliana*, genome version June 2009, and click the TAIR9 mRNA data set from the Bioviz Quickload Data source under the Data Access tab in the browser. The ESTs data used in this study are available from the Bioviz DAS2 data source, labelled "spliced EST." When users click the IGB icon (an IGB link) displayed on an ArabiTag Web page, IGB (if already running) then scrolls and zooms to the requested Difference Region. At that point, users can click the Refresh Data button to load all spliced ESTs and gene models that overlap the current region in view.

**Figure 7:**
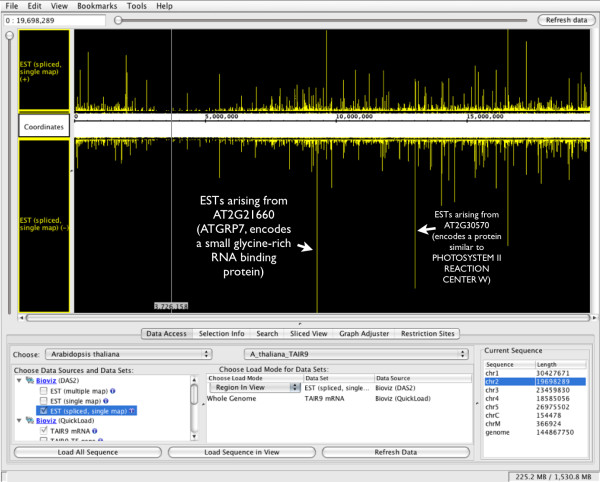
**Integrated Genome Browser showing the entire spliced EST data set for chromosome 2**. Here, the user has set the spliced ESTs track to allow maximum expansion, thus allowing the ESTs to occupy as much vertical space as necessary to show all ESTs mapping to chromosome two, even for regions with many hundreds overlapping ESTs arising from highly-expressed genes. Two of the largest EST stacks are from AT2G21660 and AT2G30570, encoding a small, glycine-rich RNA binding protein (ATGRP7) and a protein similar to photosystem II reaction center W, respectively.

By clicking a spot in the display to focus zooming and then adjusting horizontal and vertical sliders, users can zoom in on individual ESTs or gene models. Right-clicking (or control-clicking) a selected gene model or EST will open a menu giving the option to view the corresponding Web page at TAIR http://arabidopsis.org or dbEST (NCBI). Zooming all the way out (using the horizontal slider at the top of the display) provides an overview of the most abundantly represented genes on the chromosome (Figure 7). By default, because the number of overlapping ESTs for some genes is very large, IGB draws ESTs on top of each other when the total number of ESTs overlapping the same location in the genome exceeds a default value. Users can force the viewer to display arbitrarily large stacks of ESTs (as in Figure 7) by adjusting the IGB vertical display settings, as described in the IGB user's guide. Briefly, users right-click (or control-click) the track labels, select the "Adjust Max Expand" option, and enter "0" instead of the default value of ten. (The default value of ten instructs IGB to display EST stacks no taller than ten ESTs.)

## Discussion

The major result from this study is that alternative splicing in *Arabidopsis *is highly skewed: for most genes with abundant EST coverage, the currently available ESTs support one dominant splicing pattern. As illustrated in Figure 5, the bias against the minor form becomes more apparent when we limit consideration to genes with greater levels of EST coverage across the Difference Regions. Since the EST sample size is likely to correlate with overall expression levels, this bias against minor forms may be particularly acute among highly-expressed genes, as discussed in another study that used ESTs to assess alternative splicing in SR and SR-related genes in Arabidopsis [27]. Nonetheless, our study found a significant number of genes where alternative forms are prevalent and unlikely to represent sequencing artifacts. These trends in the existing data suggests that as more ESTs are sequenced, additional minor forms will appear, but that these will occur rarely when compared to the dominant isoforms.

Pending the availability of further data, we propose three possible interpretations for our results. The "noisy splicing" explanation is that for many genes with documented alternative forms, the minor, less-abundantly expressed variant represents noise in the system, i.e., errors in splicing that do not typically play an important role in regulation of gene function. Under optimal conditions, these aberrant forms may be eliminated by RNA quality control mechanisms, but when the organism is under stress, these mechanisms may be compromised, thus allowing aberrant forms to be expressed. These aberrant forms might allow for expression of phenotypic variability, some of which might aid in adaptation to the environmental stresses that allowed their production. (This idea is inspired in part by research investigating Hsp90 and its role in suppression of phenotypic variability in diverse species [28].)

Another explanation, the "cell type specific" explanation, is that the minor variants observed in the ESTs collection represent splicing variants that occur at high frequency in a very small subset of cells in *Arabidopsis*. According to the cell type specific explanation, the dominant isoform may prevail in the majority of cells and cell types, but there may in addition be some cell types where the minor isoform predominates. This is perhaps a more attractive hypothesis, in that it fits well with observations from other systems in which splicing plays a role in cellular differentiation, notably, development of the nervous system in mammals. We attempted to test this idea by searching for cell type-specific splicing choices among ESTs from the only large library in the EST collection derived from a single cell type (ovules), but found no such examples. However, the number of sequences obtained from this library may not have been sufficiently large enough to demonstrate cell-type specific splicing. If further sequencing were done on this cell type, it is possible that ovule-specific splicing patterns might emerge. However, there is also evidence supporting the noisy splicing hypothesis. For example, it is well known that splicing regulation employs quality control mechanisms (e.g., nonsense-mediated decay, reviewed in [29]) that work to eliminate aberrant splicing products and that similar mechanisms operate in *Arabidopsis *[30-32]. Of course, it is possible that both hypotheses are true for different subsets of genes subject to AS in *Arabidopsis*. Resolving these questions may become possible once we have many more EST data sets arising from single cells or from conditions that might activate noisy splicing, e.g., abiotic stresses or disease.

A third explanation is that both the minor and major forms play a role in modulating gene function and that they co-occur in the same cells. Although the majority of AS genes in *Arabidopsis *appear to favor one dominant splicing pattern, there are clearly some (but not many) genes for which AS occurs frequently and possibly even in the same cells. These genes (listed in Table 2) seem particularly likely to utilize AS as a mechanism to modulate gene function; however, it is important to note that in many cases (11/23), the affected regions occur in the 3-prime or 5-prime UTRs. In addition, most of the Difference Regions that impact the protein-coding sequence involve small changes. Taken together, these observations suggest that for the majority of *Arabidopsis *genes in which alternative splicing forms appear at high frequency, alternative splicing does not directly or dramatically affect the protein sequence but may instead exert its effects (if any) primarily at the level of RNA, such as by differential inclusion of motifs involved in controlling RNA stability, localization, or translation. However, a full assessment of this hypothesis awaits publication of more ESTs data covering more genes.

Another important result from the study is that intron retention is indeed abundant and widely supported among the ESTs. The spliced (intron removed) variant is often the minor form in AS scenarios involving intron retention. If intron retention were an aberration or an experimental artifact, we would expect to see that the vast majority of AS events involving retained introns would favor the intron-spliced (non-retained) form. But this is not the case; we found nearly one third of AS Events involving intron retention favored the intron retained form. Thus, this study agrees with other studies that investigated intron retention using other methods and came to the same conclusion: alternative splicing involving intron retention is a real phenomenon and not an artifact.

To aid biologists investigate the relative prevalence of individual splicing choices as represented in the currently available ESTs for *Arabidopsis*, we provide an on-line tool (ArabiTag) that allows users to search for splicing evidence related to individual gene models or, more broadly, perform data-mining experiments to find entire sets of genes with varying levels of overall support for the minor or major forms. To further help users investigate individual genes, we provide embedded links to the Integrated Genome Browser from within ArabiTag report pages, allowing users to click an "IBG link" and quickly navigate to the genome region affected by alternative splicing. The design of IGB allows users to view hundreds, even thousands of ESTs at once; indeed, one can even load the entire EST data set for a single chromosome. To our knowledge, this feature is unique; none of the on-line tools we tested were able to display all the ESTs for abundantly expressed loci such as the gene encoding rubisco activase. Another contribution of this work is that ArabiTag includes quality control filters necessary to be confident in an individual EST's level of support for a given intron or splicing event. Because many minor forms are only weakly supported in the ESTs (as described above), it is important to eliminate ambiguous alignments, such as from ESTs that map onto multiple genomic locations, so that users do not confuse flawed alignments data with bona fide examples of rare variants.

## Conclusions

Alternative splicing in *Arabidopsis *is highly skewed in the sense that one variation in splicing patterns tends to predominate among the majority of genes subject to AS. However, a small, but significant minority of genes appear to produce alternative transcript forms in about equal amounts, strongly suggesting that for these genes, the AS variants are co-expressed.

## Methods

### Computational Definition of AS

This study required a way to translate biological concepts of alternative splicing into algorithms suitable for addressing AS prevalence. In the TAIR9 annotations, the majority of genes have an associated AGI (*Arabidopsis *Genome Initiative) locus code, such as AT1G04231. Each locus code may have one or more associated gene models, corresponding to the mature, spliced mRNA transcripts. Gene models are identified using AGI locus codes and a numeric suffix of the form .N, where N is an integer greater than or equal to one. We identified locus codes with multiple associated transcripts (gene models) and then examined their intron-exon structures to determine if the gene models arising from the same locus exhibit alternative splicing patterns relative to each other. For this, we compared exons and introns between gene models, searching for cases where an intron in one model overlapped an exon in another model and where the region of overlap was internal to both. (The requirement that the overlapping region be internal to both models avoids mistaking alternative promoter or alternative poladenylation patterns as alternative splicing.) For each instance of an intron in one gene model overlapping an exon in another, we designate the intron as i_overlap _and the exon as e_overlap _and their two gene models respectively as Gene Absent (abbreviated G_A_) and Gene Present (abbreviated G_P_). For each i_overlap_, we also identify the intron in the other model that overlaps it; this is designated i_alt_, for alternative intron. (Alternative splicing choices involving retention of introns have no i_alt_.) The segment of genomic sequence where the intron and exon overlap is designated the Difference Region (abbreviated D_R_). Thus, the Difference Region D_R _defines a location where the two models G_A _and G_P _differ; it is a segment of sequence the G_P _model includes as part of its mature spliced sequence and which the G_A _model omits. (See Figure 1.) Note that this scheme does not attempt to categorize the overall structure of splicing for a given pair of transcripts, but instead focuses on removal of individual introns.

### Computational Analysis of AS

We aligned approximately 1.5 million *Arabidopsis thaliana *ESTs from dbEST against the *Arabidopsis *genome using blat [21]. We then filtered the alignments, retaining only those alignments with 95% identity or better across 90% of the query length. ESTs that aligned to multiple locations in the genome under these criteria were not included in subsequent analyses, leaving a data set consisting of ESTs that mapped to one, unique location in the *Arabidopsis *genome. We designate this data set the EST (single map) data set, since it includes ESTs that map onto one location in the genome. Next, we applied a third filter that identified EST alignments that showed evidence of splicing, adapting a protocol described on the UCSC Genome Informatics Genome Browser site [33] used to create its spliced EST data track, except that we allowed non-canonical splice boundaries. Briefly, any alignment that contained a gap in the EST larger than or equal to thirty bases was included. We designate this the EST (spliced, single map) data set. Both the EST (single map) and EST (spliced, single map) data sets are available via the Integrated Genome Browser under the Bioviz DAS2 data source.

Using custom python scripts, we then compared the inferred intron/exon boundaries in the ESTs with the overlapping gene models from the TAIR9 gene structure annotations. For an AS choice to be considered as supported by an EST, we required that the EST's genomic alignment contain a gap that perfectly matched the proposed boundaries of either the i_overlap _or its alternative i_alt_. For an EST to support the retention of intron, its alignment had to contain at least 20 aligned bases on either side of the intron's donor or acceptor site.

### Statistical Analysis

All statistical analyses were performed using the R statistical programming environment. For the splice choice bias analysis, we modeled each D_R _as representing two mutually exclusive choices represented by G_A _and G_P_. Each EST that supported either choice was considered as a single trial, analogous to a coin toss. For each AS event and its associated ESTs, we tested the null hypothesis that the probability of making one or the other choice was equal to 1/2, equivalent to testing whether the coin is fair. Our alternative hypothesis was two-sided, i.e., the coin was not fair. We then tested every D_R _and adjusted the resulting p values using the highly conservative Bonferroni family-wise error rate correction. The original unadjusted p values for each binomial test appear on the ArabiTag Web site at http://transvar.org/arabitag.

### Intron Support and Intron Position

To estimate bias with respect to intron position and support levels per intron, we adapted a procedure originally described in [22]. For each gene model that contained an intron, we created a spliced gene model (*g*_*s*_) and divided the spliced model into 20 regions of fifty base pairs per region, starting from the three and five prime ends of the spliced transcript for all spliced transcripts 2000 bases or larger. For each region, we counted the number of supported (*i*_*s*_) and unsupported introns (*i*_*u*_) across all the transcripts and calculated the percentage of unsupported introns per region as .

## Authors' contributions

AL conceived and directed the study. AE with assistance from AL performed programming tasks, statistical analyses, and designed the Web site. AL, KP, and AE analyzed data and designed experiments. All authors read and approved the manuscript.
